# Quantifying the Association between Bovine and Human Trypanosomiasis in Newly Affected Sleeping Sickness Areas of Uganda

**DOI:** 10.1371/journal.pntd.0002931

**Published:** 2014-06-05

**Authors:** Beatrix von Wissmann, Jenna Fyfe, Kim Picozzi, Louise Hamill, Charles Waiswa, Susan C. Welburn

**Affiliations:** 1 Division of Pathway Medicine and Centre for Infectious Diseases, School of Biomedical Sciences, College of Medicine and Veterinary Medicine, The University of Edinburgh, Edinburgh, United Kingdom; 2 Health Protection Scotland, Glasgow, Scotland, United Kingdom; 3 Department of Pharmacy, Clinical and Comparative studies, School of Veterinary Medicine and Animal Resources, Makerere University, Kampala, Uganda; IRD/CIRDES, Burkina Faso

## Abstract

**Background:**

Uganda has active foci of both chronic and acute HAT with the acute zoonotic form of disease classically considered to be restricted to southeast Uganda, while the focus of the chronic form of HAT was confined to the northwest of the country. Acute HAT has however been migrating from its traditional disease focus, spreading rapidly to new districts, a spread linked to movement of infected cattle following restocking. Cattle act as long-term reservoirs of human infective *T. b. rhodesiense* showing few signs of morbidity, yet posing a significant risk to human health. It is important to understand the relationship between infected cattle and infected individuals so that an appropriate response can be made to the risk posed to the community from animals infected with human pathogens in a village setting.

**Methodology/Principal Findings:**

This paper examines the relationship between human *T. b. rhodesiense* infection and human infective and non-human *T. brucei* s.l. circulating in cattle at village level in Kaberamaido and Dokolo Districts, Uganda. The study was undertaken in villages that had reported a case of sleeping sickness in the six months prior to sample collection and those villages that had never reported a case of sleeping sickness.

**Conclusions and Significance:**

The sleeping sickness status of the villages had a significant effect with higher odds of infection in cattle from case than from non-case villages for *T. brucei* s.l. (OR: 2.94, 95%CI: 1.38–6.24). Cattle age had a significant effect (p<0.001) on the likelihood of *T. brucei* s.l. infection within cattle: cattle between 18–36 months (OR: 3.51, 95%CI: 1.63–7.51) and cattle over 36 months (OR: 4.20, 95%CI: 2.08–8.67) had significantly higher odds of *T. brucei* s. l. infection than cattle under 18 months of age. Furthermore, village human sleeping sickness status had a significant effect (p<0.05) on the detection of *T. b. rhodesiense* in the village cattle herd, with significantly higher likelihood of *T. b. rhodesiense* in the village cattle of case villages (OR: 25, 95%CI: 1.2–520.71). Overall a higher than average *T. brucei* s.l. prevalence (>16.3%) in a village herd over was associated with significantly higher likelihood of *T. b. rhodesiense* being detected in a herd (OR: 25, 95%CI: 1.2–520.71).

## Author Summary

Human African trypanosomiasis (HAT) is a fatal parasitic disease of humans. Domestic cattle act as reservoirs for *T.b. rhodesiense* HAT. In a survey of cattle in Ugandan villages; six that had a reported HAT case in the previous six months, and six which did not, an association was detected between presence of HAT in a village and infection of village cattle with human infective parasites. Village HAT status was significant (p<0.05) for detection of *T.b. rhodesiense* in the village herd, with significantly greater likelihood of *T.b. rhodesiense* in herds from case villages (OR:25,95%CI:1.2–520.71). *T. brucei* s.l. prevalence of over 16.3% in cattle was associated with significantly greater likelihood of *T.b. rhodesiense* infection within the herd (OR:25, 95%CI:1.2–520.71). Cattle age had a significant effect (p<0.001) on the likelihood of a *T. brucei* s.l. infection: cattle between 18–36 months (OR:3.51,95%CI:1.63–7.51) and over 36 months (OR:4.20,95%CI:2.08–8.67) had significantly higher rates of *T. brucei* s.l. than animals under 18 months of age. Cattle are long-term reservoirs of *T.b. rhodesiense* that will outlive control strategies that do not directly eliminate parasite infection. *T.b. rhodesiense* and/or elevated *T. brucei* s.l. prevalence in village herds can serve as an indicator for HAT risk.

## Introduction

Tsetse transmitted trypanosomiasis contributes a significant burden for both human and animal disease across tsetse infested Sub-Saharan Africa. African animal trypanosomiasis (AAT), manifests as a spectrum of diseases, most commonly caused by *Trypanosoma congolense*, *T. vivax* and *T. brucei* s.l., with the former two species being more pathogenic [1]. AAT is widely considered to be among the most important diseases limiting agricultural output in affected areas [2], [3]. Human African trypanosomiasis (HAT) exists as two distinct subspecies of *T. brucei* s.l.; the chronic form of HAT, caused by *Trypanosoma brucei gambiense*, is found in western and central Africa, with the acute form of the disease, caused by *T. b. rhodesiense*, found in east and southern Africa [4]. In the past, both *T. b. rhodesiense* and *T. b. gambiense* have given rise to large scale epidemics claiming many thousands of lives [5], [6]. In recent years the number of cases reported to the World Health Organization (WHO) has been steadily declining to a fifty year low of 10,000 cases in 2009 [7]. Despite this significant progress, HAT remains a serious public health problem in many areas of sub-Saharan Africa. Under-reporting remains a problem in many endemic areas exacerbated by poor health infrastructure, lack of public awareness, and civil conflict leading to the breakdown of disease monitoring and control [8] so that the true burden and impact of HAT is difficult to calculate. Recent estimates suggest 70 million people live in areas at risk of contracting HAT [9].

**Figure 1:**
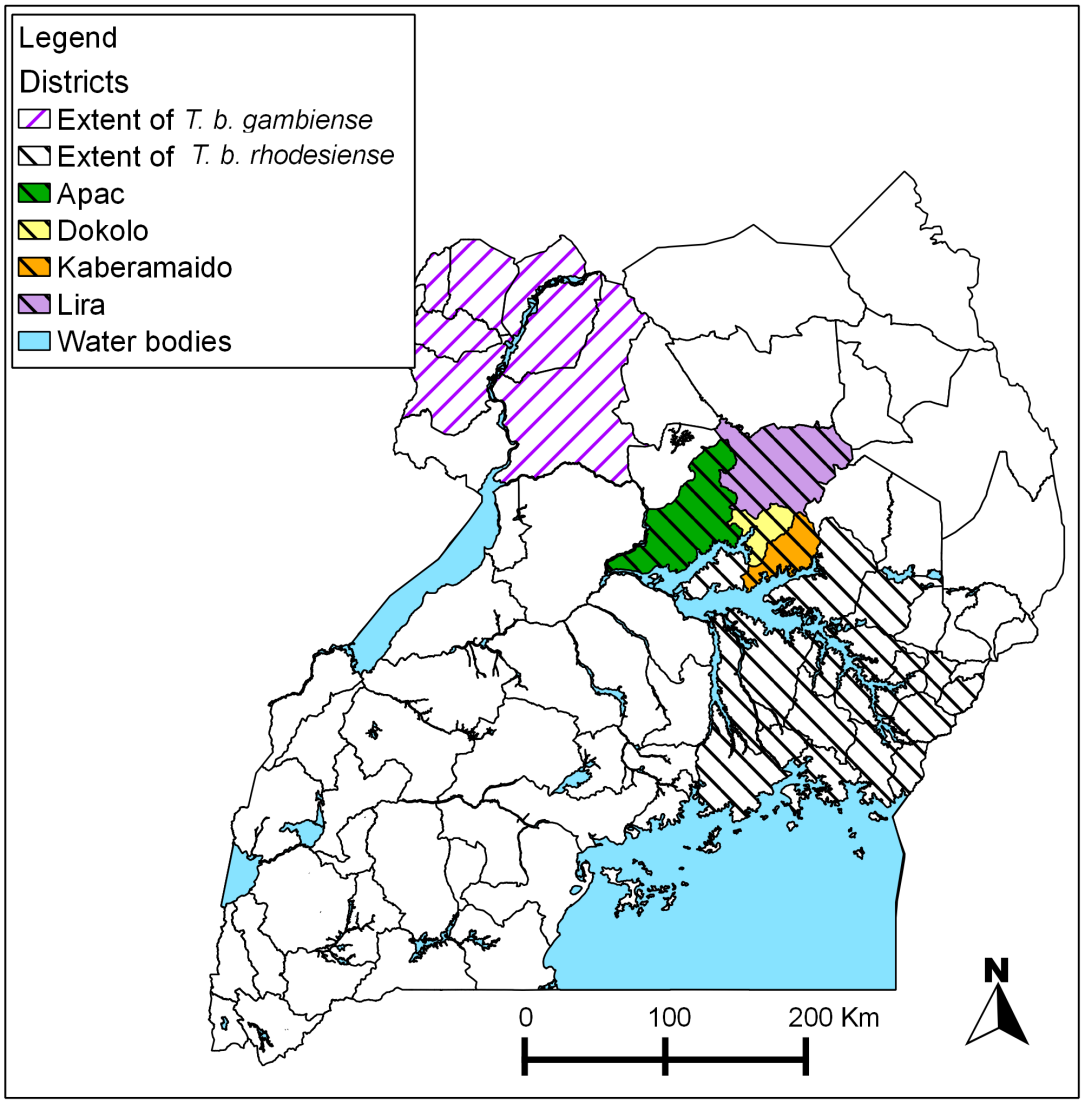
Map of Uganda showing the location of study districts Kaberamaido, Dokolo, Apac and Lira.

Uganda has active foci of both chronic and acute HAT within its borders. Until recently the acute form was confined to southeast Uganda, while the focus of the chronic form was confined to the northwest of the country. In recent years the two disease foci have been converging and in 2005 were separated by only 150 km[10]. Since 2000, acute HAT has spread to six previously unaffected districts of Uganda putting an additional 1 million people at risk[11], [12], [13]. There are 32 districts in Uganda that have reported cases of acute or chronic HAT since 2000 [7], as shown in figure 1. Consequently these districts can be considered high risk for human sleeping sickness, nine of these account for 80% of all reported Rhodesian sleeping sickness cases in the country. Several factors are implicated in this spread but especially significant is the movement of cattle northwards from areas endemic for HAT and animal trypanosomiasis [14], [15] - cattle co-infected with both human-infective *T. b. rhodesiense* and parasites that cause AAT. Movement of such cattle has introduced parasites to districts formerly free of human disease but where tsetse are present and able to transmit disease. In Tororo District 23% of cattle were found to be infected with *T. b. rhodesiense* [16] as were 18% of cattle being traded in Soroti District [17]; cattle were shown to be the main reservoir of infection for humans in this area [18].

At the start of the outbreak in this previously unaffected area, HAT cases were clustered around the local cattle market, which was trading a high proportion of cattle sourced from endemic *T. b. rhodesiense* areas [11], [15], [19]. Government policy now requires all cattle to be treated with trypanocides at point of sale [20], but this has not prevented the continuing spread of disease along the northern shores of Lake Kyoga and surrounding swampland. In addition to the threat to an increasing number of people, the continuing northward spread of Rhodesian sleeping sickness raises the prospect of an overlap between Rhodesian and Gambian foci. If the two forms of the disease become sympatric, diagnosis and treatment of sleeping sickness, already problematic, will be compromised as diagnosis and treatment regimes differ for the two infections [17], [21], [22].

Since the first human cases were detected in Soroti in 2001, cases of Rhodesian sleeping spread to neighbouring districts; >500 cases have been reported from Kaberamaido District and the neighbouring Dokolo District since 2004. Analysis of the spatial distribution of HAT cases within the newly affected districts of Dokolo and Kaberamaido showed that proximity to a cattle market was a significant risk factor for human infection with *T. b. rhodesiense* [23]. However there is little information on the prevalence of trypanosomiasis in cattle in these districts, despite their important role as a reservoir for human infective parasites. Decentralisation of the veterinary services in Uganda and budgetary restrictions, have resulted in animal trypanosomiasis no longer considered a priority disease in Uganda and surveillance being suspended.

This study aims to investigate the extent of the reservoir of human infective *T. b. rhodesiense* in cattle in order to provide an indicator of risk to the human population in the affected districts, as well as to permit appraisal of the risk of the disease spreading to neighbouring districts. To examine the prevalence and distribution of *T. brucei* s.l. (both of non-human infective *T. b. brucei* and human infective *T. b. rhodesiense*) in village cattle, samples were taken from villages that had reported a case of sleeping sickness in the six months before sample collection (defined as case villages) and from those that had never reported a case of sleeping sickness (defined as non-case villages). This approach allowed a comparison of *T. brucei* s.l. prevalence and presence or absence of *T. b. rhodesiense* in cattle between case and non-case villages.

## Materials and Methods

### Sample sites

Two hospitals, Serere hospital in Soroti and Lwala hospital in Kaberamaido, were equipped to diagnose and treat cases of sleeping sickness from across the study area comprising Apac, Dokolo, Kaberamaido and Lira (see Figure 1). All diagnosed cases from Kaberamaido, Dokolo, Lira and Apac are referred to one of these two treatment centres. At the beginning of July 2006 villages were identified from which a case of HAT had originated during the previous six-month period, these villages were defined as ‘case villages’.

**Figure 2:**
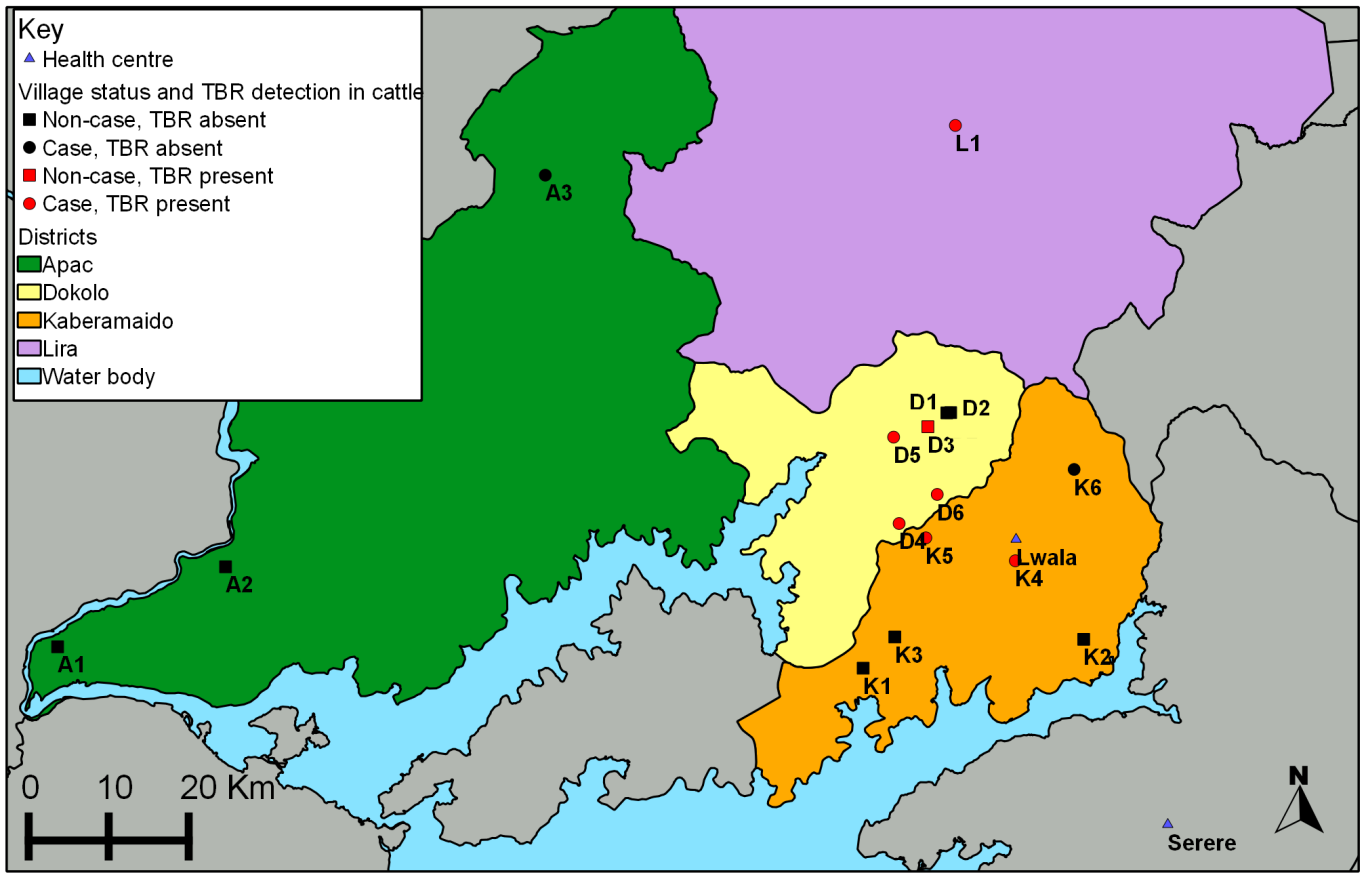
Location of sampling sites: Village cattle were sampled at three case village sites and three non-case villages sites each in Kaberamaido and Dokolo Districts (non- case villages: K1–3, D1–3; case villages: K4–6, D4–6) as well as 4 additional villages in Lira and Apac Districts (non-case villages: A1–2; case villages L1, A3). Case village are shown as circles, non-case villages are shown as squares, those villages from which *T. b. rhodesiense* was detected in cattle are coloured red. *T. brucei* s.l. prevalence of respective villages provided in Table 1.

Village cattle were sampled at three case village sites and three non-case village sites each in Kaberamaido and Dokolo Districts (Figure 2). The six non-case villages were randomly selected from a list of villages located in neighbouring parishes to the selected case villages within the respective two districts, to ensure similar environmental conditions whilst avoiding overmatching [24]. The Euclidian distance between any two villages included in the study ranged from a minimum of 0.5 km to a maximum of 38.4 km, with the average distance between villages being 19.5 km. At the time of sampling, non-case villages had never reported a case of HAT (since the first case of Rhodesian HAT was reported in Kaberamaido District in 2004).

In addition to the 12 study villages from Kaberamaido and Dokolo, cattle from 4 further villages of interest were sampled. An additional case village was selected in Aloi sub-county, Lira District (village L1), the sub-county furthest to the north in Lira District to report HAT cases in 2005. In Apac District village cattle were sampled from the only village that had reported a case of sleeping sickness (village A3, Figure 2) and from 2 non-case villages proximal to the only operating cattle market in Apac (villages A1 and A2, Figure 2).

The required sample size was estimated based on the sample required for comparing two proportions (case vs. non-case villages), with adjustment for the village cluster design [24]. A total sample size of 1000 to 1200 animals was estimated as the required sample size, including adjustment for clustering at the village level (intra-cluster correlation coefficient at the village herd level estimated as 0.12, unpublished; 80-100 animals in each of the 12 study villages), to allow a 20 percent-point difference in trypanosome prevalence (5% v 25%) to be detected between case and non-case villages (95% confidence, 80% power). In each village between 80-100 cattle blood samples were taken, systematically by order of presentation of the animals at the central site, unless fewer than 80 cattle attended the sampling, in which case all cattle present at the central site were sampled. Permission was sought from individual owners to sample their cattle and from the District Veterinary Officers (DVOs) of each study district. For each sampled animal, age group was recorded as identified by owner or by tooth eruption pattern (category a: milk teeth, under 18 months of age; category b: one pair of permanent incisors, between 18months and 3 years of age; category c: more than one pair of permanent incisors, over 3 years of age).

Geographic coordinates of each sampling site were recorded using a hand held Global Positioning System (GPS, Garmin) to allow mapping of their exact location (Figure 2).

### Sample collection

Blood was drawn from the ear vein of each sampled animal into heparinised capillary tubes and applied immediately onto an FTA card (Whatman, Maidstone, Kent, UK). FTA cards were left to dry and placed together with desiccant in airtight multi-barrier pouches (Whatman, UK).

Additional information recorded for each animal sampled included cattle breed, age, sex and body condition category.

### Laboratory analyses

Five 3 mm discs were punched from each blood sample and processed in preparation for PCR as previously described [25]. In short, these discs were washed a total of four times, twice with FTA purification reagent and twice with TE buffer, before being air dried and then heated at 90°C for thirty minutes while suspended in a 5% (w/v) chelex solution. Five mircoliters of this extract was used to seed a PCR targeting *T. brucei* s. l. [26]. Samples positive for *T. brucei* s. l., were further tested by PCR specific for the human infective subspecies *T.b. rhodesiense* [17]. Standard PCR amplifications for all reactions were carried out in 25 µl mixtures. PCR reaction conditions, primer sequences and adapted cycling conditions were previously published in [27]. One positive control [genomic deoxyribonucleic acid (DNA)] and one negative control (extract from blank FTA disc) were run with each reaction. PCR products were resolved via electrophoresis on 1.5% agarose gels stained with ethidium bromide and run at 100 v for a minimum of forty-five minutes in the presence of a molecular marker, until band size could be easily determined.

### Statistical analysis

Prevalence of *T. brucei* s. l. as detected by PCR was expressed as a percentage, and exact binomial 95% confidence intervals were computed (R, version 2.0.1). All further statistical analysis (uni- and multivariable mixed models) was restricted to the 12 study villages (6 case villages, 6 non-case villages) in Kaberamaido and Dokolo. The effect of the study design was estimated by including village herd as a random effect in a model without fixed effect predictors, and the percentage of total variance occurring at the level of the random effect was estimated using the latent variable approach [24].

The effect of cattle age group, condition category (categorical variable: poor, moderate, good), gender (binary variable: male/female), breed (categorical variable: zebu, ancholi, zebu-ancholi-cross), village administrative district (binary variable: Kaberamaido/Dokolo) and village sleeping sickness status (binary variable: case/non-case) on the odds of *T. brucei* s. l. infection were initially examined at the univariate level including village herd as a random effect, followed by step-wise forward construction of a multivariable level mixed model, considering any factor significant at the 80% level (p≤0.2) in the univariable analysis. Factors significant at the 95% level (p≤0.05) were retained in the multivariable mixed model. A final mixed effect model with village as random effect, to account for village level variation, and cattle age, cattle condition category and case/non-case status of each village as fixed effects was constructed.

Since presence of *T. b. rhodesiense* was of interest at the village herd level, the village herd was used as the epidemiological unit for analysis. District, village sleeping sickness status (binary variable: case/ non-case status), herd age structure, herd condition category (binary variable: less/ more than 10% of herd in poor condition), herd age structure (binary variable: less/ more than 20% of herd in youngest age category) were investigated using univariable logistic regression. Apart from sleeping sickness status, no variable was significant at the 80% level, thus no multivariable model was constructed.

## Results

In total 1428 cattle were sampled from 16 villages and of these, 221 were infected with *T. brucei* s. l., as detected by PCR (15.5%; 95%CI: 13.7–17.4%). Across all the villages sampled, fifteen of the animals that were observed to be infected with *T. brucei* s.l were harbouring *T. b. rhodesiense* human infective parasites (1.05%, 95% CI 0.59–1.72%). The number of *T. brucei* s. l. and *T. b. rhodesiense* positive samples across the 12 study villages within the main sleeping sickness focus in Kaberamaido and Dokolo, as well as the 4 additional villages of interest from Lira and Apac are shown in Table 1. Using the latent variable approach [24] for a model fitted with village herd as the random effect and no fixed effects other than the intercept, the proportion of the variance occurring at the herd level in the 12 study villages in Kaberamaido and Dokolo was estimated to be 39.6%. Due to a considerable proportion of the variance being explained at the herd level, herd was included as random effect in subsequent analyses.

**Table 1:** The number of *T. brucei* s. l. and *T. b. rhodesiense* positive samples in each of the 12 main study villages and the 4 additional villages of interest.

District	Sleeping sickness status	Village ID	No. of samples	No. of *T. brucei* s. l. positives	Proportion of *T. brucei* s. l. positives (%)	No. of samples positive for *T. b. rhodesiense*
**Main study villages**						
Kaberamaido	non-case	K1	95	4	4.2	0
		K2	83	5	6.0	0
		K3	100	17	17.0	0
	case	K4	80	14	17.5	2
		K5	92	28	30.4	1
		K6	70	3	4.3	0
Dokolo	non-case	D1	68	5	7.4	0
		D2	63	2	3.2	0
		D3	104	16	15.4	2
	case	D4	99	29	29.3	5
		D5	100	20	20.0	2
		D6	100	29	29.0	2
Total (Study villages)			1054	172	16.3	14
**Additional villages of interest**						
Lira	case	L1	100	12	12.0	1
Apac	non-case	A1	80	2	2.5	0
		A2	95	3	3.2	0
	case	A3	99	32	32.3	0
Total (Additional villages of interest)			374	49	13.1	1
**Total (Overall)**			1428	221	15.5	15

### Analysis of *T. brucei* s. l

The effect of individual risk factors on the odds of *T. brucei* s. l. infection and modeled as univariable fixed effect (with village herd included as the random effect) considered for inclusion in the multivariable mixed effect model is shown in Table 2, based on the 12 study villages in Dokolo and Kaberamaido. The sleeping sickness status of the villages had a significant effect with higher odds of infection in cattle from case than from non-case villages (OR: 2.94, 95%CI: 1.38–6.24, p<0.01). Cattle age also had a significant effect (p<0.001) on the odds of *T. brucei* s. l. infection. Cattle between 18–36 months (OR: 3.51, 95%CI: 1.63–7.51, p<0.01) and cattle over 36 months (OR: 4.20, 95%CI: 2.08–8.67, p<0.001) showed significantly higher odds of *T. brucei* s. l. infection than did younger cattle that were under 18 months of age. Condition score was also considered as a variable for inclusion in the multivariable mixed effect model (p = 0.131. Cattle that were observed to be in good condition had significantly lower odds of having a *T. brucei* s. l. infection detected as compared to cattle that were considered to be in poor condition (OR: 0.48, 95%CI: 0.23–0.98, p<0.05). Cattle in moderate condition also had lower odds of having a *T. brucei* s. l. infection detected than cattle in poor condition but this was not statistically significant (OR: 0.65, 95%CI 0.37–1.12, p = 0.118).

**Table 2:** Effect of individual risk factors as univariable fixed effect, (as presented by crude OR, CI and univariable p-value) with herd included as the random effect, and in final multivariable mixed effect model (as presented by adjusted OR, CI and p-value).

Variable	Factor levels	Total (n = 1054)	*T. brucei* s. l. positives (%)	Crude odds ratio (OR)	95% confidence interval crude OR	Univariable p-value	Adjusted OR	95% CI adjusted OR	Adjusted p-value
**Village Sleeping Sickness status**									
	Non-case	513	49 (9.6)	1			1		
	Case	541	123 (22.7)	2.94	1.38–6.24	<0.01	2.96	1.40–6.26	<0.01
**Age Category**						**<0.001**			**<0.001**
	A	168	9 (5.4)	1			1		
	B	256	43 (16.8)	3.51	1.63–7.51	<0.01	3.86	1.79–8.32	<0.001
	C	630	120 (19.0)	4.2	2.08–8.67	<0.001	4.6	2.24–9.45	<0.001
**Condition category**						** = 0.131**			**<0.05**
	Poor	89	21 (23.6)	1			1		
	Medium	826	133 (16.1)	0.65	0.37–1.12	= 0.118	0.68	0.39–1.18	= 0.169
	good	139	18 (12.9)	0.48	0.23–0.98	<0.05	0.4	0.19–0.82	<0.05

NB: Bolded p-values are likelihood ratio test p-values and non-bolded p-values are Wald test p-values.

Uni- and multivariable mixed effect models are based on the 12 main study villages.

Village sleeping sickness status (p<0.01), cattle age (p<0.001) and cattle condition score (p<0.05) were retained as fixed effects in the final multivariable model for the 12 study villages in Dokolo and Kaberamaido (summarised in Table 2). Village herd was included as the random effect in the final models to account for the hierarchical structure of the data; 23.8% of the total variance occurred at village herd level.

### Analysis of human infective *T. b. rhodesiense*

Amongst the 12 study villages in Kaberamaido and Dokolo, village sleeping sickness status had a significant effect on the detection of *T. b. rhodesiense* in cattle belonging to the village herd, with significantly higher odds of *T. b. rhodesiense* being detected in the village herd from which a human sleeping sickness case had been reported (case village) (OR: 25, 95%CI: 1.2–520.71, p<0.05).

A higher than average *T. brucei* s. l. prevalence within the village herd of more than 16.3%, was also associated with significantly higher odds of *T. b. rhodesiense* being detected in the respective herd (OR: 25, 95%CI: 1.2–520.71, p<0.05). No other variable was significant at the 80% level. Due to the high degree of co-linearity between sleeping sickness status of the village and higher than average *T. brucei* s. l. prevalence (see Table 1), these two factors were not considered in a multivariable model.

## Discussion

Previous studies have suggested an association between human and bovine trypanosomiasis in areas of Uganda where *T. b. rhodesiense* has long been established [23], [28] indicating the potential importance of the cattle reservoir in transmission of Rhodesian sleeping sickness [11], [17], [29]. The results presented here demonstrate a clear link between outbreaks of sleeping sickness in a village and the prevalence of *T. brucei* s. l. and *T. b. rhodesiense* in the cattle of that village herd. Establishing the extent of the reservoir of human infective *T. b. rhodesiense* parasites in cattle not only provides an indicator of risk to the human population in the affected districts but also permits appraisal of the risk of the disease spreading to neighbouring districts.

In the present work we found a significant association between sleeping sickness status of a village with the odds of *T. brucei* s.l. infection in the village cattle herd. Amongst the 12 study villages in Kaberamaido and Dokolo, *T. brucei* s.l. prevalence was higher in herds from case than from non-case villages. This association remained significant when adjusted for cattle age category and condition score, which were significantly associated with the odds of *T. brucei* s. l. infection; these associations are well described in the literature [30], [31], [32] but did not act as confounders. Case villages were significantly more likely to have cattle carrying *T. b. rhodesiense* in the village herd suggesting a highly focal association between presence of the parasite in local cattle, and the risk of transmission to the human population of the respective villages.

Including the 4 additional villages of interest, *T. b. rhodesiense* was detected in cattle in six out of eight case village herds, and in one out of eight of the non-case village herds. It is possible that *T. b. rhodesiense* was only recently introduced to the non-case village herd where human transmission has not yet been observed or that underreporting of sleeping sickness [8] may have led to misclassification of the sleeping sickness status of this village. *T. b. rhodesiense* was found at lower levels within cattle in Kaberamaido and Dokolo Districts than in the sleeping sickness endemic areas further to the south east, where the ratio of *T.b. rhodesiense* to *T. b. brucei* infections in cattle was relatively stable at 1∶3 [33]. The comparatively low prevalence of the human infective *T. b. rhodesiense* in Kaberamaido and Dokolo may be attributable in part to its apparently recent introduction to these areas. Analysis of the distribution of human sleeping sickness cases in newly affected districts has shown evidence for the dispersal of Rhodesian sleeping sickness into areas with more ‘suitable’ ecology over time, and diminishing spatial association with the initial point of introduction [13]. Mathematical modelling of the transmission dynamics (including any competitive advantages between trypanosome species and sub-species) would be required to investigate whether, in the absence of interventions, the prevalence of *T. b. rhodesiense* in newly affected areas would be expected to reach endemic levels over time, as the distribution of the pathogen converges with its optimal ecological niche. The low prevalence of *T. b. rhodesiense* detected in the cattle in Kaberamaido and Dokolo Districts none-the-less promotes transmission to humans as indicated by the 338 cases of *T. b. rhodesiense* sleeping sickness reported by the end of June 2006.

Awareness of HAT in the newly affected Districts of Dokolo and Kaberamaido is low. Cases of human trypanosomiasis are often confused with other diseases, such as malaria or HIV/AIDS by the local population, and a doctor will not necessarily be consulted due to lack of funds and the long distances from health facilities. Lwala hospital, in Kaberamaido District, located close to the border to Dokolo District is the only health facility in the area that provides routine diagnosis and treatment for HAT. A very high proportion of cases are diagnosed in the late stage of the disease and there is significant under-reporting. The level of under detection in newly affected districts will be at least as high, if not higher, than in other parts of Uganda, where for every person that is reported to die of sleeping sickness, another 12 die of the disease undetected [8]. Even in areas where HAT has been present for many years, the disease is frequently misdiagnosed [34], so even when awareness of HAT increases in Dokolo and Kaberamaido, under-reporting will likely remain a significant problem. Reported cases represent the tip of an iceberg with respect to people infected with HAT.

Even though village cattle herds from the most northern sleeping sickness case village in Lira at the time of sampling (village L1) showed only a moderate prevalence of *T. brucei* s.l. (see Table 2), *T. b. rhodesiense* was detected in the village herd, again providing evidence of a local reservoir of this human infective parasite in village livestock.

Mapping of reported cases has shown a continued northward spread of the disease within Dokolo and Lira District and in the summer of 2006 the first case of sleeping sickness was reported from Apac District [13], [22] (additional village of interest A3). However, district officials indicated that the infected individual had spent time away from their home village, in Soroti District, where sleeping sickness is endemic so it is by no means certain that zoonotic transmission had been established in Apac. *T. brucei* s.l. prevalence in the two non-case villages (A1 and A2) sampled in Apac was low at under 4% in both villages and no *T. b. rhodesiense* was detected. However, a high prevalence of *T. brucei* s.l. was observed in the case village herd (A3 in Figure 2) in Apac District although no *T. b. rhodesiense* was detected in this village herd. Whilst no *T. b. rhodesiense* was detected in any of the cattle samples from Apac, the *T. brucei* s. l. prevalence in the cattle herd of the case village (A3) was observed to be as high or higher than the prevalence detected in case villages in Kaberamaido and Dokolo that is indicative of HAT risk.

The present study showed a strong association between high levels of *T. brucei* s. l. in a village herd, the likelihood of detecting *T. b. rhodesiense* and the status of the village as a sleeping sickness case village. This could provide a useful means of determining risk at village or parish level in endemic districts in Uganda. A high village level prevalence of *T. brucei* s.l. could be used to predict risk for human infection and justify herd level chemotherapy to remove circulating human infective (and other animal infective) parasites from the herd, given the specificity and sensitivity of molecular tools that are available for diagnosis of *T. brucei* s.l. [35].

It is clear from the close link between animal and human infection at village level that any sustainable intervention against sleeping sickness should be targeted and sustained at local community level. Control of infection in the animal reservoir through appropriate veterinary intervention with effective cross talk between relevant Ministries could realise the value of a One Health Approach for zoonotic disease control [36].
